# Supplementary material for: Translational control in the spinal cord regulates gene expression and pain hypersensitivity in the chronic phase of neuropathic pain
Source: eLife. 2026 Apr 10;13:RP100451. doi: 10.7554/eLife.100451 (PMC13068433; doi:10.7554/eLife.100451)
Supplement: Figure 2—source data 1. [file elife-100451-fig2-data1.zip › Figure_2,_Source_Data_1.pdf]

Figure 2B

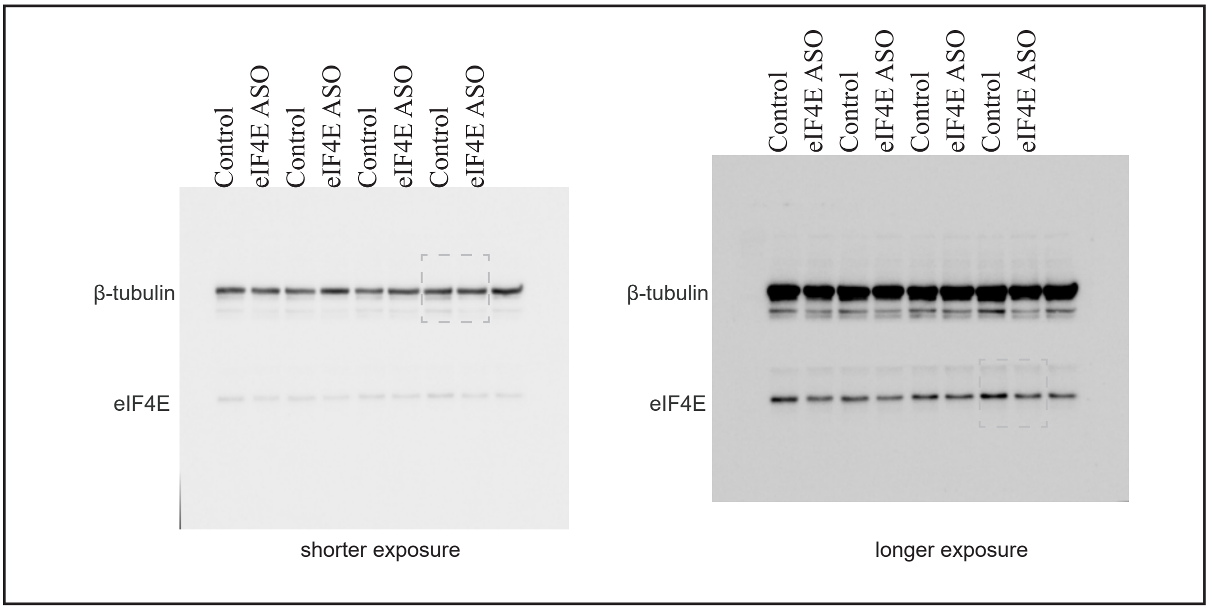

Figure 2C

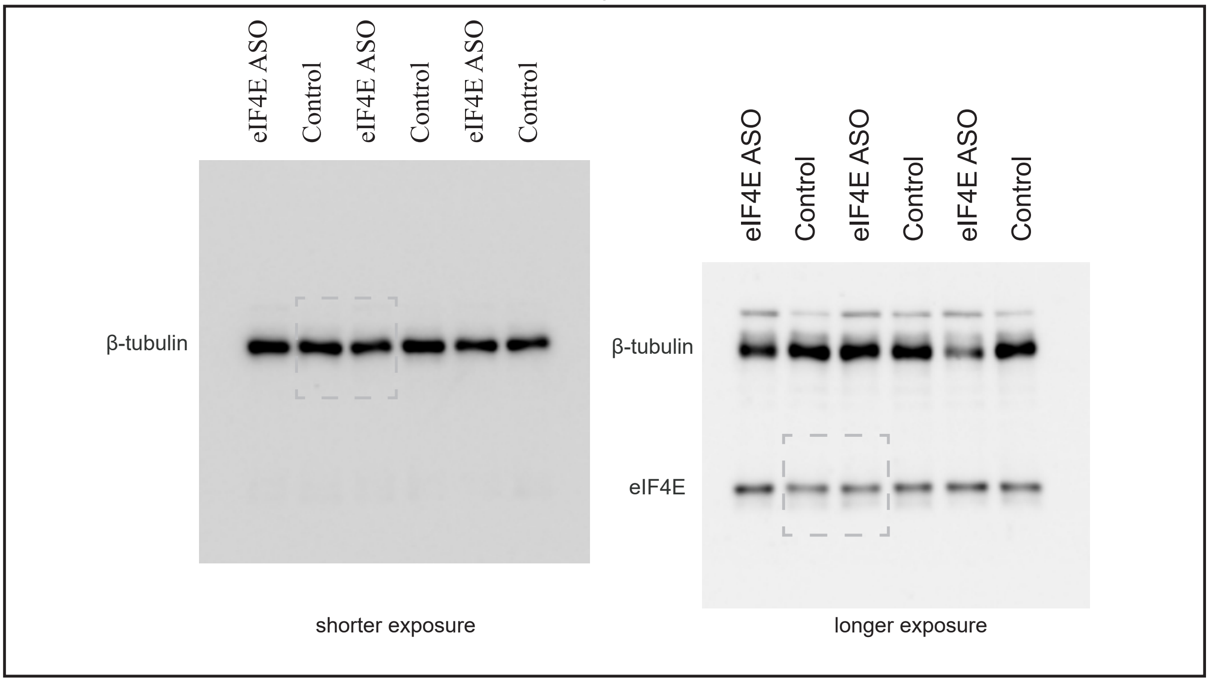

Figure 2-Source Data 1. Original membranes corresponding to Figure 2. Upper blots correspond to panel B, and lower blots correspond to panel C. Shorter exposures are shown on the left, and longer exposures on the right. The presented bands are marked by dashed squares.
