# Supplementary figures and images for: Translational control in the spinal cord regulates gene expression and pain hypersensitivity in the chronic phase of neuropathic pain
Source: eLife. 2026 Apr 10;13:RP100451. doi: 10.7554/eLife.100451 (PMC13068433; doi:10.7554/eLife.100451)

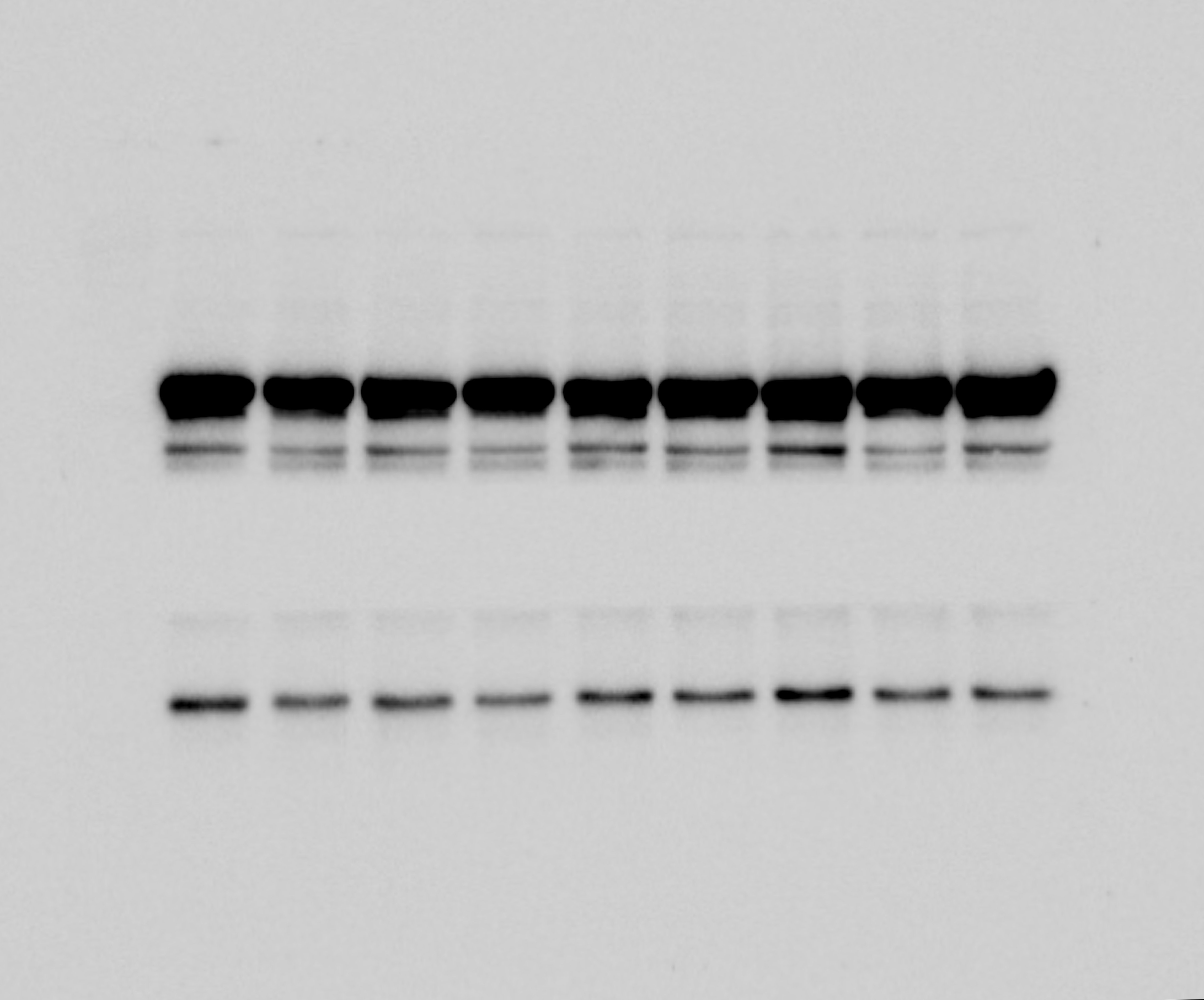

Supplement: Figure 2—source data 2. [file elife-100451-fig2-data2.zip › Figure 2B long exposure.jpg]

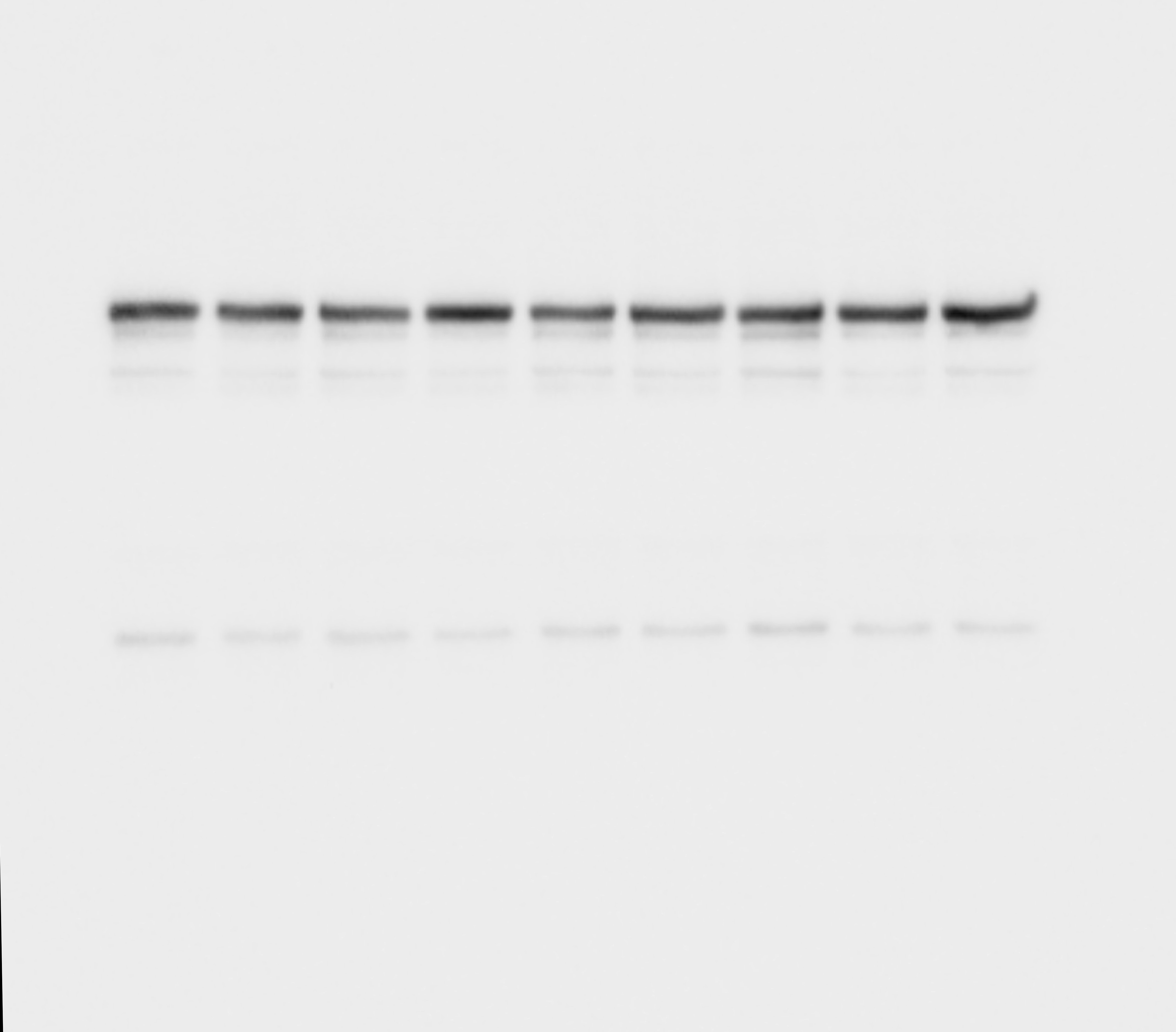

Supplement: Figure 2—source data 2. [file elife-100451-fig2-data2.zip › Figure 2B short exposure.jpg]

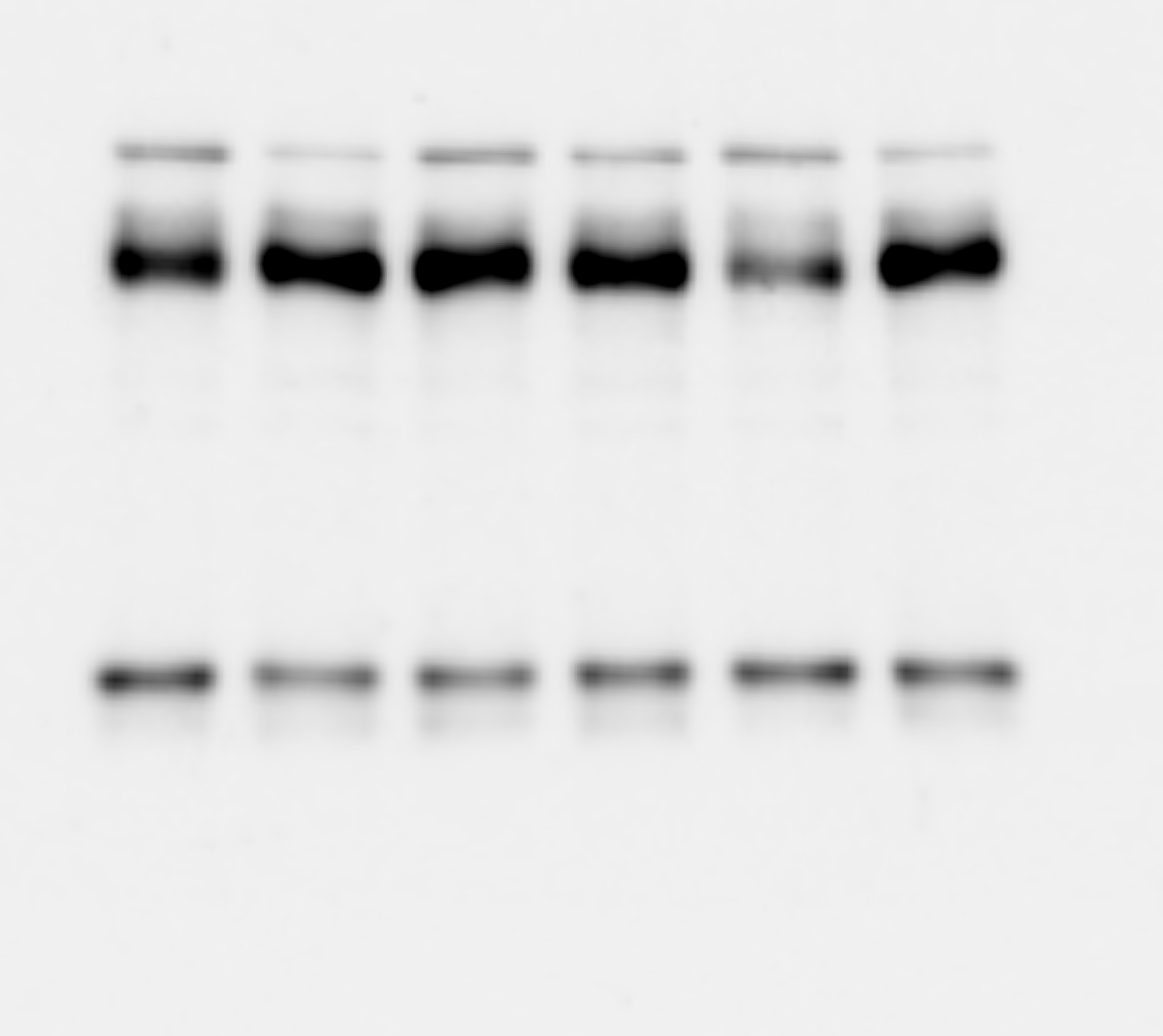

Supplement: Figure 2—source data 2. [file elife-100451-fig2-data2.zip › Figure 2C long exposure.jpg]

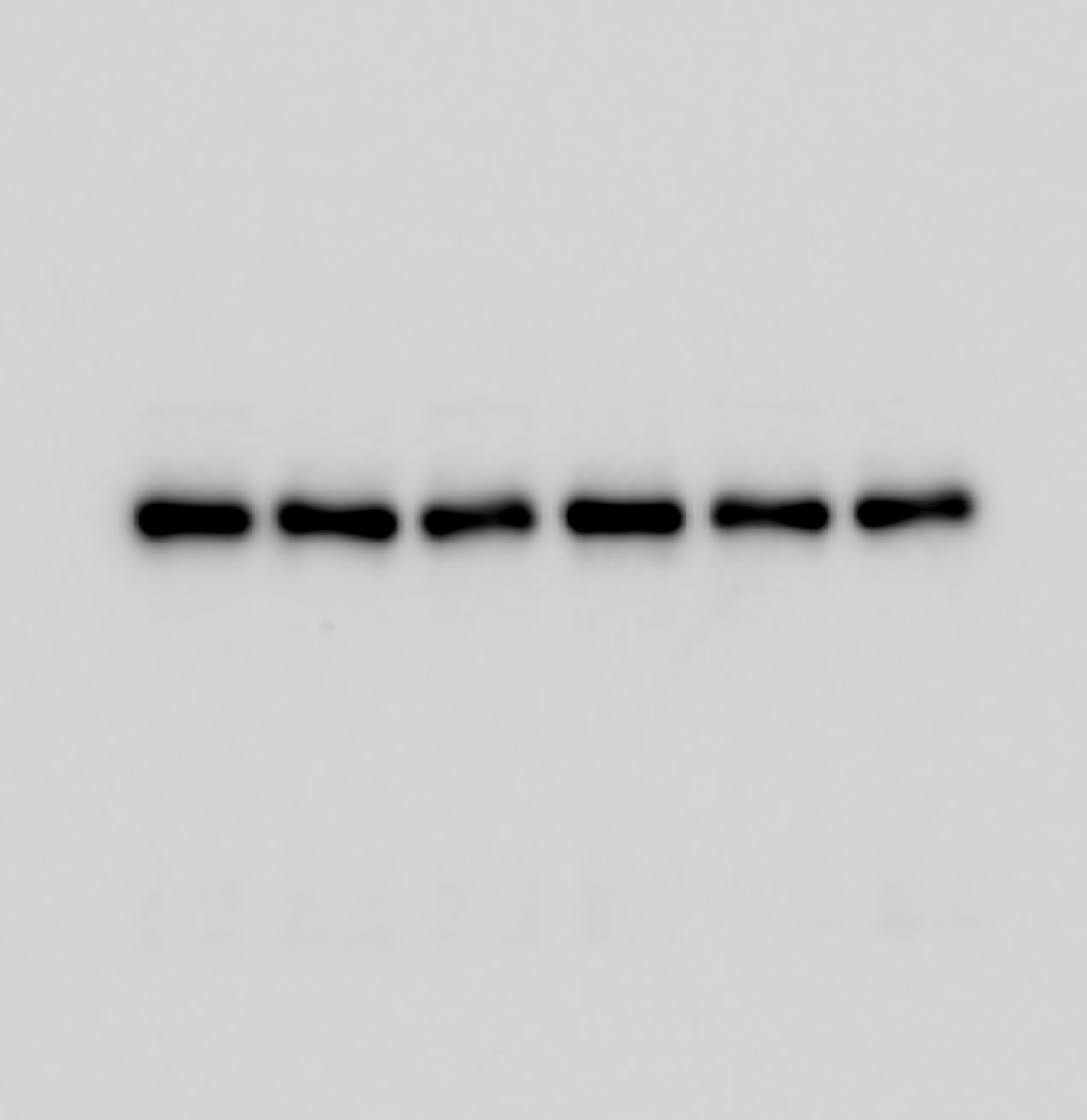

Supplement: Figure 2—source data 2. [file elife-100451-fig2-data2.zip › Figure 2C short exposure.jpg]
